# Primary medical care in Irish prisons

**DOI:** 10.1186/1472-6963-10-74

**Published:** 2010-03-22

**Authors:** Joe M Barry, Catherine D Darker, David E Thomas, Shane PA Allwright, Tom O'Dowd

**Affiliations:** 1 Department of Public Health & Primary Care, Trinity College, Dublin, Ireland; 2 College Health Service, Trinity College, Dublin, Ireland

## Abstract

**Background:**

An industrial dispute between prison doctors and the Irish Prison Service (IPS) took place in 2004. Part of the resolution of that dispute was that an independent review of prison medical and support services be carried out by a University Department of Primary Care. The review took place in 2008 and we report here on the principal findings of that review.

**Methods:**

This study utilised a mixed methods approach. An independent expert medical evaluator (one of the authors, DT) inspected the medical facilities, equipment and relevant custodial areas in eleven of the fourteen prisons within the IPS. Semistructured interviews took place with personnel who had operational responsibility for delivery of prison medical care. Prison doctors completed a questionnaire to elicit issues such as allocation of clinician's time, nurse and administrative support and resources available.

**Results:**

There was wide variation in the standard of medical facilities and infrastructure provided across the IPS. The range of medical equipment available was generally below that of the equivalent general practice scheme in the community. There is inequality within the system with regard to the ratio of doctor-contracted time relative to the size of the prison population. There is limited administrative support, with the majority of prisons not having a medical secretary. There are few psychiatric or counselling sessions available.

**Conclusions:**

People in prison have a wide range of medical care needs and there is evidence to suggest that these needs are being met inconsistently in Irish prisons.

## Background

The use of primary health care in the prison population is considerable compared with the general community [1]. Health needs of prisoners are diverse and complex [2]. Prisoners are more preoccupied with their health than the general population [3,4]. Relative to the general population, prison inmates experience poorer physical, mental and social health, including both acute and long standing physical and mental illness and disability, drug, alcohol and tobacco dependency, sexual health problems, suicide, self -harm, physical, psychological and sexual violence, lower life expectancy and breakdowns in family and other relationships [5-8]. Hepatitis C has been shown to be endemic in Irish prisons [9] and substance misuse is an underlying problem for a large proportion of prisoners [10]. In the UK, prisoners consult their GP three times more than the demographically equivalent population in the community [11]. There are fourteen prisons across the Republic of Ireland, catering for both males and females, ranging from open centres to closed high security facilities. The most recent annual report of the Irish Prison Service [12] indicates that the number of committals to prison increased from 10,658 in 2005 to 12,157 in 2006, an increase of 14.1 percent. The Irish Prison Service (IPS) provides medical care in Irish prisons. The IPS recognises the importance of healthcare and rehabilitation "in sustaining prisoners' physical and mental health, counteracting the detrimental effects of imprisonment and encouraging positive personal development from within" [13]. The General Healthcare Study of the Irish Prisoner Population [14] found that when prisoners were compared with the general population, they reported lower levels of physical and mental health and had higher lifetime polydrug use, including higher levels of alcohol consumption and cigarette smoking. However, their diet was comparable and exercise patterns were better and their blood pressure readings were lower than that of the general population.

Medical care is coming under increasing pressure to address issues such as equivalence of care (whereby prison medical services should provide the same quality and range of care provided in the wider community [15]); concerns regarding the quality of medical facilities and expediting medical administration duties. There is a dearth of international literature focusing on medical facilities and infrastructure within the prison system and of what impact these may have on the provision of medical care to the prison population.

We were commissioned by the IPS, with the agreement of the Irish Medical Organisation, to carry out a review of structural and support services in primary care in Irish prisons. This arose as a consequence of an industrial dispute between prison doctors and the Irish Prison Service. We have used the data from that review to describe here primary medical care infrastructure in the IPS.

## Methods

### Study setting

Eleven of the 14 prisons in the Republic of Ireland serving over 78% of the total prison population were visited during the time period from November 2007 to February 2008. The prisons included in the study were one high security closed prison, nine medium security closed prisons and one open prison. Three prisons, two open (one of which houses young offenders) and one medium security prison (which houses sex offenders), were excluded from the study due to funding reasons as the review budget did not allow for 14 prison visits. The three excluded prisons were excluded on the basis that other prisons of similar function were included in the sample.

### Measures

A questionnaire was devised by the authors and circulated to all prison doctors prior to the review visit (Additional File 1). This sought information on specific training, allocation of clinicians' time, continuous professional development, computerisation, security, nurse and administrative support and overall resources available.

A checklist was completed by one of the authors (DT) based on the instrument used by the assessors in the Competence Assurance Exercise in general practice carried out under the auspices of the Medical Council in 2007 (Additional File 2). One of the authors (DT) had been an assessor for that exercise. The key areas were based on those proposed by Frazer [16] and included the state of premises, equipment, contracted hours and time allocation for various tasks and support staff.

### Procedure

Escorted review of the medical facilities, equipment and relevant custodial areas was undertaken by one of the authors (DT). On the day of the visit, semi-structured interviews were carried out with senior prison management, the prison doctor and the prison nursing team. The interview covered the operation and delivery of medical services within the prison.

### Ethics approval

The chair of the ethics committee of the Irish College of General Practitioners was contacted in relation to this study. The study was granted exemption from ethics approval as patients were not approached by the researchers.

## Results

### State of premises

The medical unit infrastructure provided up until recently in the majority of Irish prisons dated from the mid-nineteenth century. Facilities in six prisons were deemed unsuitable and facilities in five prisons deemed adequate for provision of modern primary medical care by the inspector (DT) (Table 1).

**Table 1:** Medical facilities within the Irish Prison Service at time of inspection November 2007 - February 2008.

Prison	Construction date	Medical facilities description	Modern medical facilities		Suitability of facilities
			**Planned**	**Built, but not in use at time of inspection**	

1	Built as a psychiatric hospital in 1930 and converted for prison use in 1996	One surgery and one interview/office; no waiting area; unsuitable for number of medical staff	Refurbishment planned 2009		No
2	Opened 1999	Well appointed facilities			Yes
3	Opened1814. Refurbished 1983; modified 1989	Totally inadequate for modern primary care delivery	No improvements planned		No
4	Opened 1999	Adequate for present use			Yes
5	Opened 1821	Single, windowless room with no modern facilities; allowed use of adjoining facilities	Opened March 2008		Yes
6	Opened 2000	Four consultation rooms; treatment room; methadone administration room.			Yes
7	Opened 1850. Refurbished 2006	Adequate facilities; underutilised due to operational difficulties			Yes
8	Opened 1840	Totally inadequate for modern primary care delivery	Due for expansion	Due to open 2008	No
9	Opened 1973	Reasonably well appointed; requires refurbishment	Due for minor refurbishment		Yes
10	Opened 1958	Totally inadequate; 1 single room	Minor refurbishment & enlargement due Jan 2009		No
11	Opened 1989	Totally inadequate for present needs.	Due for expansion of services	To be commissioned Feb 2009	No

### Equipment

Based on the national survey the "Structure of General Practice in Ireland 1982-2005" [17] the availability of standard diagnostic equipment in Irish prisons generally falls below that available in Irish general practice (Table 2).

**Table 2:** Comparison of availability of medical equipment in Irish prisons with availability in Irish general practice

Prison	Opthalm- oscope	Peak Flow	Auto-clave	ECG	Defib + resuscitate trolley	Spirometer
1	Yes	Yes	No	No	Yes	No
2	Yes	Yes	No	No	Yes	No
3	Yes	Yes	No	No	Yes	No
4	Yes	Yes	No	No	Yes	No
5	Yes	No	No	No	Yes	No
6	Yes	Yes	No	No	Yes	No
7	Yes	Yes	No	No	Yes	Yes
8	Yes	Yes	No	Requested	Yes	No
9	Yes	Yes	Yes	No	Yes	No
10	Yes	Yes	No	No	Yes	No
11	Yes	Yes	No	Yes	Yes	No
						
% Irish prisons	100%	90%	9%	9%	100%	9%
% Irish general practices*	99%	97%	88%	77%	37%	55%

ECG = electrocardiogram

* Source: 'Structure in general practice in Ireland 1982-2005' (O'Dowd, O'Kelly, O' Kelly, 2006)

### Staff contracted hours and methadone dispensing services

The ratio of prisoners to doctor contracted time was calculated (Table 3). Half of the prisons had ten or more prisoners per doctor hour. Doctors reported that nearly 50% of their time was spent on committals and transfers, with most reporting doing their own administrative duties. Seven prisons have methadone dispensing facilities. The various types of support staff working within the health care system were also documented.

**Table 3:** Ratio of the number of prisoners to prison doctor contracted hours in prisons, support staff and methadone dispensing available within the IPS.

Prison	Prison population	Doctor contract hours per week	Prisoners/doctor hour	Nurse WTE	Secretary WTE	Medical orderly WTE	Psychiatry sessions per week	Counsellors	Methadone dispensing
1	239	15	15.9	7	0	0	1	2	No
2	433	55	7.8	18	1	0	6	2 sanctioned	Yes
3	280	15	18.6	0	0	6	3		No
4	85	22.5	3.7	8	Shared	1	3		Yes
5	284	15	18.7	5	0	5	2		No
6	450	78	5.7	11	2 hours	1	2		Yes
7	556	78	7.1	14	1	14	5		Yes
8	178	15	11.8	4	0	2	1		Yes
9	58	10	5.8	10	0	1	0		Yes
10	217	15	14.5	4	0	2	3		No
11	370	39	9.5	15	0	2	4	2 WTE	Yes

WTE = whole time equivalent

### Support staff

The availability of other members of the health care team such as nurses, secretaries, medical orderlies, psychiatrists and counsellors was examined. Although most prisons had some nursing staff at the time of visit, one prison had none. The majority of prisons did not have a medical secretary, had few medical orderlies, few available psychiatric sessions, and limited numbers of counsellors (Table 3). The whole time equivalent (WTE) data given in Table 3 was provided by the Irish Prison Service. It is an administrative term calculated by management to take account of the fact that not all staff work full time. There were nursing managers in four of the prisons visited with further nurse manager appointments imminent. It is hoped that this will provide leadership for the nursing staff and a much needed career structure.

The IPS relies heavily on medical orderlies to support medical care delivery within the prisons. Their current role includes providing security surveillance, night cover and response to emergency situations such as stabbings, cuttings and cardiac arrest. In effect, medical orderlies have a paramedical type role together with medical administrative duties.

## Discussion

This is the first national study of primary medical care infrastructure and facilities within the Irish Prison Service based on an independent inquiry. There were broad differences between prisons in standards of medical care. These differences arose from wide variations in the infrastructure of medical units; availability of basic medical equipment, doctor-prisoner ratios, the scope of services provided and the levels of support from ancillary services.

### Limitations of study

There are a number of limitations to the study. In the first instance only one inspector was used. The study was carried out in the context of a commissioned review of primary care structures and support services and the review budget allowed for only one inspector. On the other hand, this ensured comparability of inspections at all prison sites. Secondly, not all of the information given in the doctors' questionnaire could be corroborated. Where it could be it was. Where it could not be or where it contradicted official data this was checked with the prison authorities at a series of meetings that were held as part of the review. Thirdly, there were other results, which were open to interpretation, in particular decisions as to what was or was not deemed "suitable". Ultimately, the inspector had to make a global judgement based on all the evidence presented. For these reasons the results in this study may not be reproducible or applicable in other jurisdictions.

### State of facilities

The United Nations' standard minimum rules for treatment of prisoners states, "where hospital facilities are provided in an institution, their equipment, furnishings and pharmaceutical supplies shall be proper for the medical care and treatment of sick prisoners" (recommendation 22.2 [18]). The medical unit infrastructure provided up until now in the older establishments within the IPS dates from Victorian or pre-Victorian times and is inadequate for the provision of good quality modern medical care. Imprisonment presents opportunities for health promotion and health improvement in a generally "hard to reach" population [19]. However, the antiquated facilities currently found in the IPS limit the service that medical staff can provide. This was previously highlighted in the list of specific deficiencies drawn up by the Irish Medical Organisation and in reports, such as the Council of Europe [20] report on the 'Prevention of Torture and Inhuman or Degrading Treatment or Punishment'. However, a new medical block has been commissioned in one prison, and refurbishment is expected to take place in three other prisons imminently. When fully operational, the new facilities will equate favorably with facilities in modern multidisciplinary primary medical care units within the community.

### Equipment

There is no international benchmark for the exact types and quantities of medical equipment that should be present within a prison medical unit. The recommendation from the Council of Europe [21] to member states on the European Prison Rules suggests "where a prison service has its own hospital facilities, they shall be adequately staffed and equipped to provide the prisoners referred to them with appropriate care and treatment" (Recommendation 46.2). It is difficult to adopt the notion of equivalence of care within an Irish context as there are currently no Irish national standards for equipment in general practice surgeries. However, the type of medical equipment provided within the IPS appears to be below that normally available in a 'General Medical Services' public scheme practice in the community.

### Contracted hours and time allocation for various tasks

The range of clinician times across prisons given in Table 3 means that there is currently an imbalance in the provision of medical services. There is a need for a clear benchmark regarding the ratio of doctors to prisoners within the Irish prison context. Both the Council of Europe report [20] and the General Healthcare Study of the Irish Prisoner Population [14] stressed the immediate need to increase the numbers of full time equivalent doctors in a number of Irish prisons in order to achieve the objective of equivalence of care. However, the proportion of time that doctors spend on committals and transfers of prisoners is approximately half of their working day. This work comprises mostly fuller assessment on entry of a prisoner to a given prison. The time spent on other clinical duties is much lower than the estimated contracted time.

### Support staff

Until 1999 there were no nurses employed in the IPS [22]. Nurses working within the IPS are working in custodial environments and this has led to some confusion about the boundaries of their role [23]. Nurses carry out a number of non-nursing duties such as clerical and administrative work and escort duties. It has been reported that there is a conflict between the 'divergent aims' [24] of correctional officers and nurses due to different 'underlying assumptions' of providing health care on the one hand and correction on the other.

While medical orderlies continue to provide a valuable service, their present role needs to be redefined. They are suitably placed to provide the much needed administrative and secretarial duties, while maintaining their escort duties and chaperoning doctors and nurses when the need arises. There is a need for medical administrative support within the service. There are only two full time dedicated medical secretaries employed in the IPS. Consideration could be given to the retraining and redeployment of experienced medical orderlies to fill this important role. This would lead to increased efficiency within the medical units and prevent the practice whereby prison doctors, for practical reasons, are taking administrative work off-site.

### Equivalence of care

Prisoners retain the right, as set out in the United Nations declaration, to have medical care equivalent to that available to those outside prison [15]. However, measuring performance of medical care in a prison against that provided under the 'General Medical Services' scheme in the community is particularly difficult in an Irish context. Currently, the IPS is responsible for providing medical services in Irish prisons [25], rather than the integrated systems adopted in Norway, France and the United Kingdom (UK) where prison medical care is contracted out to agencies equivalent to the Irish Health Services Executive (HSE) [26,27]. The Department of Health in the UK assumed responsibility from Her Majesty's Prison Service for health policy in 2000 and full budgetary and health care administration control were transferred by 2006. As a result of this reorganisation, resources and funding have improved and services now relate more to assessed health needs. UK governmental health strategies, such as 'Choosing Health' [28] now also include prisoners as a special target group in relevant national initiatives such as smoking cessation programmes and combating blood borne viruses programmes [29]. Notwithstanding these changes there are still many challenges in delivering quality healthcare in the prison setting [30]. The IPS can learn from experiences in the United Kingdom in this regard. In particular, similar improvements could be gained if the IPS adopted a population health approach and encouraged health promotion initiatives. Transferring responsibility for the delivery of medical services to the Health Services Executive may possibly best facilitate this.

## Conclusions

To improve medical care, policies inside prison need to be consistent with those outside [31]. People in prison have a range of medical care needs and there is evidence to suggest that these needs are being met inconsistently in Ireland. The ratio of doctor-contracted time to the number of prisoners varies widely between prisons. The question of the types and quantity of medical equipment that should be available within the IPS remains unanswered. Thus, the medical care provided currently within the IPS is variable. People in prison are part of the wider community that prisons serve; it remains inappropriate that health care is provided differently within the prison and the community. In order to move from a "prison medical service" to a "prison health service", a significant change in the ethos of the IPS is required with more emphasis on prevention and health promotion.

## Competing interests

The authors declare that they have no competing interests.

## Authors' contributions

All authors participated in the design and conception of the study. DET collected the data. All authors participated in the analysis and coordination and helped to draft the manuscript. All authors read and approved the final manuscript.

## Pre-publication history

The pre-publication history for this paper can be accessed here:

http://www.biomedcentral.com/1472-6963/10/74/prepub

## Supplementary Material

Additional file 1 **Doctors Questionnaire**. A self-administered questionnaire by the prison doctors. Click here for file

Additional file 2 **Facility and equipment checklist**. Completed by one of the authors (DT). Click here for file
