# Supplementary material for: Primary medical care in Irish prisons
Source: BMC Health Serv Res. 2010 Mar 22;10:74. doi: 10.1186/1472-6963-10-74 (PMC2853535; doi:10.1186/1472-6963-10-74)
Supplement: Additional file 1 — Doctors Questionnaire. A self-administered questionnaire by the prison doctors. [file 1472-6963-10-74-S1.DOC]

**Appendix A**

DOCTORS’ QUESTIONNAIRE

| Name of doctor: |  |  | |
| --- | --- | --- | --- |
|  |  |  | |
|  |  |  | |
| Name of prison: |  |  | |
|  |  |  | |
|  |  |  | |
| Length of time in position: |  | |  |
|  |  | |  |

| Are you full time? |  |  |
| --- | --- | --- |
|  |  |  |
| Are you part time? |  |  |
|  |  |  |
| How many hours per week? |  |  |
|  |  |  |
| Length of typical session? |  |  |
|  |  |  |
| In a typical session what percentage age of time is spent on: | Routine GP type consultations |  |
|  |  |  |
|  | Committals/transfers  /discharge examinations |  |
|  |  |  |
|  | Administration and reports |  |
|  |  |  |
| In an typical hour what is the number of routine GP consultations you would be required to see |  |  |
|  |  |  |
|  |  |  |

**Computerisation**

| **Please rate 1 Very Poor 2 Poor 3 Fair**  **4 Good 5 very good 6 Excellent**  **U/A Unable to assess** |  | **1** | **2** | **3** | **4** | **5** | **6** | **U/A** |
| --- | --- | --- | --- | --- | --- | --- | --- | --- |
|  |  |  |  |  |  |  |  |  |
| How do you rate the PMRS as a multidisciplinary record system? |  |  |  |  |  |  |  |  |
|  | |  |  |  |  |  |  |  |
| How do you rate the Prison Health Information System as a tool for the recording of primary care records? | |  |  |  |  |  |  |  |
|  |  |  |  |  |  |  |  |  |
| Are you familiar with any other computerised GP records? If so how do you rate them: |  |  |  |  |  |  |  |  |
|  | Health 1 |  |  |  |  |  |  |  |
|  |  |  |  |  |  |  |  |  |
|  | GP Clinical/Dynamic |  |  |  |  |  |  |  |
|  |  |  |  |  |  |  |  |  |
|  | Socrates |  |  |  |  |  |  |  |
|  |  |  |  |  |  |  |  |  |
|  | Other |  |  |  |  |  |  |  |

How do you feel the system could be improved?

**………………………………………………………………………………………………………**

**………………………………………………………………………………………………………**

**………………………………………………………………………………………………………**

**………………………………………………………………………………………………………**

**Persona**l **Securit**y

| **Please rate 1 Very Poor 2 Poor 3 Fair**  **4 Good 5 very good 6 Excellent** | |  | | 1 | 2 | | 3 | 4 | 5 | | 6 |
| --- | --- | --- | --- | --- | --- | --- | --- | --- | --- | --- | --- |
| How do you rate security within the Medical Unit? | |  | |  |  | |  |  |  | |  |
|  |  | |  | | |  | | | |  | |
|  |  | | Never | | | Occasionally | | | | Frequently | |
| Have you felt threatened in the course of your work within the Medical Unit? |  | |  | | |  | | | |  | |
|  |  | |  | | |  | | | |  | |
| How many times have you felt threatened over the past year? |  | |  | | |  | | | |  | |
|  |  | |  | | |  | | | |  | |
|  |  | | Never | | | Occasionally | | | | Frequently | |
| Have you ever been physically assaulted in the course of your work within the Medical Unit? |  | |  | | |  | | | |  | |
|  |  | |  | | |  | | | |  | |
| How many times have you been assaulted in the past year? |  | |  | | |  | | | |  | |

How do you feel the system could be improved?

………………………………………………………………………………………………………

………………………………………………………………………………………………………

………………………………………………………………………………………………………

………………………………………………………………………………………………………

**Nursin**g **Assistanc**e

Please circle one number on each scale, from 3 (all the time) to 0 (not at all), to indicate how often you feel each statement has applied to your practice in the past few weeks

All Not

the at

time all

In a typical session is a nurse available to you? 3 2 1 0

In a typical session for how long is a nurse

available to you? 3 2 1 0

What tasks does the nurse do:

Triage of GP attenders? 3 2 1 0

Phlebotomy? 3 2 1 0

Smear test (where applicable)? 3 2 1 0

STD screening ? 3 2 1 0

Assist in minor ops? 3 2 1 0

Contraceptive advice? 3 2 1 0

Inhaler advice? 3 2 1 0

Vaccination? 3 2 1 0

Dressings? 3 2 1 0

ECG? 3 2 1 0

Removal of sutures? 3 2 1 0

Ear syringing? 3 2 1 0

Assistance with clerical duties? 3 2 1 0

Escort duties to and from surgery? 3 2 1 0

How do you feel the system could be improved?

………………………………………………………………………………………………

………………………………………………………………………………………………

………………………………………………………………………………………………

………………………………………………………………………………………………

**Administrative Support**

All Not

the at

time all

In a typical session is administrative support

available to you? 3 2 1 0

In a typical session for how long is administrative

support available to you? 3 2 1 0

What tasks do the administrative officers do?

Organise the appointments 3 2 1 0

Assist in the preparing of reports 3 2 1 0

Collection of statistics 3 2 1 0

How do you feel the system could be improved?

………………………………………………………………………………………………

………………………………………………………………………………………………

………………………………………………………………………………………………

………………………………………………………………………………………………

**Resources Available To You**

Surgical referrals

| **Surgical** | On site |  |
| --- | --- | --- |
|  |  |  |
|  | Local hospital |  |
|  |  |  |
|  | Waiting times |  |
|  |  |  |
|  | No. of referrals per week |  |

| **Internal medicine** | On site |  |
| --- | --- | --- |
|  |  |  |
|  | Local hospital |  |
|  |  |  |
|  | Waiting times |  |
|  |  |  |
|  | No. of referrals per week |  |

**Outreach Forensic Psychiatric Services**

| **Psychiatrist** | On site |  |
| --- | --- | --- |
|  |  |  |
|  | If on site, how many sessions |  |

|  |  |  |
| --- | --- | --- |
|  | Local hospital |  |
|  |  |  |
|  | Waiting times |  |
|  |  |  |
|  | No. of referrals per week |  |

| **Psychiatric hospitalisation** | Access to in-patient treatment CMH |  |
| --- | --- | --- |
|  |  |  |
|  | Waiting times for in-patient CMH treatment |  |
|  |  |  |
|  | Referral to local psychiatric hospital |  |
|  |  |  |
|  | Waiting times for in-patient  Local psychiatric hospital treatment |  |
|  |  |  |
|  |  |  |

| **Dental facilities** | On site |  |
| --- | --- | --- |
|  |  |  |
|  | Local dental surgery |  |
|  |  |  |
|  | Dental Hospital |  |
|  |  |  |
|  | No. of sessions |  |
|  |  |  |
|  | Waiting times |  |
|  |  |  |
|  | No. of referrals per week |  |

| **Psychology - counselling** | On site |  |
| --- | --- | --- |
|  |  |  |
|  | Outside location |  |
|  |  |  |
|  | If on site, how many sessions |  |
|  |  |  |
|  | Waiting times |  |
|  |  |  |
|  | No. of referrals per week |  |

| **Physiotherapist** | On site |  |
| --- | --- | --- |
|  |  |  |
|  | Local physiotherapy clinic |  |
|  |  |  |
|  | Local hospital |  |
|  |  |  |
|  | Waiting times |  |
|  |  |  |
|  | No. of referrals per week |  |

| **Interpretative services for routine consultations** | Always |  |
| --- | --- | --- |
|  |  |  |
|  | Occasionally |  |
|  |  |  |
|  | Never |  |
|  |  |  |

| **Other: (please specify)** |  |  |
| --- | --- | --- |
|  |  |  |
| Do you participate in a methadone programme? | Level 1 |  |
|  |  |  |
|  | Level 2 |  |
|  |  |  |

Health Promotion

| Is there a health promotion officer available? |  | | |  |
| --- | --- | --- | --- | --- |
|  |  | | |  |
| Are you involved in health promotion design, planning or execution? | |  | |  |
|  |  | | |  |
|  |  | | |  |
| During the past 7 days, how many hours were you exposed to other people’s smoke in the prison? | | |  |  |

What areas do you see presenting health promoting issues in prison? ............................................................................................................................................................

............................................................................................................................................................

............................................................................................................................................................

………………………………………………………………………………………………………

………………………………………………………………………………………………………

Prevention

|  |  | All | Some | None |
| --- | --- | --- | --- | --- |
| At time of reception, are prisoners offered hepatitis B and C screening? |  |  |  |  |
|  |  |  |  |  |
| What arrangements are made for following up results of screening?  ……………………………………………………………………………………………………… | | | | |
|  |  |  |  |  |
| What services are provided for those with positive screen?  ……………………………………………………………………………………………………… | | | | |
|  |  |  |  |  |
| What are the arrangements for those discharged prior to results being returned?  ……………………………………………………………………………………………………… | | | | |
|  |  |  |  |  |
|  |  | All | Some | None |
| At time of reception, are prisoners offered hepatitis B vaccination? |  |  |  |  |
|  |  |  |  |  |
| Accelerated or standard schedule?  ……………………………………………………………………………………………………… | | | | |
|  |  |  |  |  |
| What arrangements are made for delivering subsequent doses?  ……………………………………………………………………………………………………… | | | | |
|  |  |  |  |  |
| Please estimate what proportion receive the full course.  ……………………………………………………………………………………………………… | | | | |
|  |  |  |  |  |
| What are the arrangements for those released prior to completion?  ……………………………………………………………………………………………………… | | | | |
|  |  |  |  |  |

Tuberculosis

| Do you screen every prisoner? | |  |  |
| --- | --- | --- | --- |
|  | |  |  |
| Do you screen only high risk prisoners? | |  |  |
|  | |  |  |
| Do you have access to an Area Medical Officer for tuberculin testing and specialist advice? | | |  |
|  | |  |  |
| In case of positive diagnosis do you: | refer to out patient clinic? | |  |
|  |  | |  |
|  | have the support of a chest physician? | |  |

Continuing Medical Education

| Do you have extra qualifications or training in aspects of prisoner health? |  | | |  |
| --- | --- | --- | --- | --- |
|  |  | | |  |
| Occupational health? |  | | |  |
|  |  | | |  |
| Forensic? |  | | |  |
|  |  | | |  |
| Psychiatry? |  | | |  |
|  |  | | |  |
| Family planning? |  | | |  |
|  |  | | |  |
| STDs? |  | | |  |
|  |  | | |  |
| Health Care Management? |  | | |  |
|  | |  | |  |
| Do you have ready access to training? | |  | |  |
|  |  | | |  |
| Do you see a role for the Association of Prison Doctors in CME? | | |  |  |
|  |  | | |  |
| Would you be interested in a Diploma in Prisoner Health? | | |  |  |
|  | | |  |  |
| Would you consider that there should be a formal link with an academic institution? | | |  |  |

Have you any suggestions for the role of CME. in prison health?

............................................................................................................................................................

............................................................................................................................................................

………………………………………………………………………………………………………

………………………………………………………………………………………………………

Have you any comments on the development of multi­disciplinary teams for primary care within the prison health system?

............................................................................................................................................................

............................................................................................................................................................

………………………………………………………………………………………………………

……………………………………………………………………………………………………… Have you any other comments or suggestions?

………………………………………………………………………………………………………

………………………………………………………………………………………………………

………………………………………………………………………………………………………

……………………………………………………………………………………………………...
