# Supplementary material for: Primary medical care in Irish prisons
Source: BMC Health Serv Res. 2010 Mar 22;10:74. doi: 10.1186/1472-6963-10-74 (PMC2853535; doi:10.1186/1472-6963-10-74)
Supplement: Additional file 2 — Facility and equipment checklist. Completed by one of the authors (DT). [file 1472-6963-10-74-S2.DOC]

**Appendi**x B **FACILITY AND EQUIPMEN**T **CHEC**K **LIS**T

Prison ....................................................................................................

Date of Inspection..................................................................................

Name of Nurse or Medical orderly …………………………………..

Number of Nurses .................................................................................

Number Medical Orderlies.....................................................................

| **Facilities** |  |  |  |
| --- | --- | --- | --- |
| Size of prison population |  |  |  |
| No. of nursing / medical interview rooms |  |  |  |
|  |  |  |  |
| **Rooms** | Yes |  | No |
| Sufficient size to accommodate three people |  |  |  |
| Examination couch |  |  |  |
| Washbasin |  |  |  |
| Secure filing cabinets for notes and confidential papers |  |  |  |
| Cleanliness |  |  |  |
| Heating |  |  |  |
| Telephone – direct line to outside |  |  |  |
|  |  |  |  |

|  |  |  |  |
| --- | --- | --- | --- |
| **Equipment** | Yes |  | No |
| Stethoscope |  |  |  |
| Auroscope and clean tips |  |  |  |
| Ophthalmoscope |  |  |  |
| Reflex hammer |  |  |  |
| Height/weight scales and measuring tape |  |  |  |
| Thermometer |  |  |  |
|  | Yes |  | No |
| Vaginal disposable speculum (in female prisons) |  |  |  |
| Disposable gloves |  |  |  |
| Lubricant |  |  |  |
| Tongue depressors |  |  |  |
| Ear syringe |  |  |  |
| Urine testing equipment |  |  |  |
| Spirometer and / or peak flow meter |  |  |  |
| ECG with interpretation |  |  |  |
| Tourniquet |  |  |  |
| Eye charts |  |  |  |
| AED and availability throughout prison and level of trained personnel |  |  |  |
| Airways laedel pocket masks and trained personnel |  |  |  |
| Sharps and storage and disposal |  |  |  |
| Clinical waste containers and disposal |  |  |  |
| Surgical instruments and dressings |  |  |  |
| Emergency equipment |  |  |  |
| Autoclave or sterilizer |  |  |  |
| Drugs and secure storage |  |  |  |
| Refrigeration for storage of vaccines and system for checking min & max temperature daily |  |  |  |
| Arrangements for blood sampling and dealing with body fluids |  |  |  |
| Laboratory facilities |  |  |  |
| Computer terminal |  |  |  |
| Computerized prisoner medical record system |  |  |  |
|  |  |  |  |
| **General Office Area** | Yes |  | No |
| Secretarial support |  |  |  |
| Fax |  |  |  |
| Photocopier |  |  |  |
| Shredder |  |  |  |
| Stationery |  |  |  |
| Computer |  |  |  |
|  |  |  |  |
|  | Yes |  | No |
| Waiting area |  |  |  |
| Toilet facilities |  |  |  |
|  |  |  |  |

Comments ................................................................................................
..................................................................................................................
..........................................................................................................................................
..........................................................................................................................................
..........................................................................................................................................
..........................................................................................................................................
..........................................................................................................................................

Signed ........................................................................................................
Dr David Thomas

**Nurse checklist:**

During surgery hours do you:

|  |  | Always | Occasionally | Never |
| --- | --- | --- | --- | --- |
| Escort patients to and from surgery |  |  |  |  |
|  |  |  |  |  |
| Set up GP appointments |  |  |  |  |
|  |  |  |  |  |
| Retrieve files |  |  |  |  |
|  |  |  |  |  |
| Attendance with GP in surgery |  |  |  |  |
|  |  |  |  |  |

While assisting the general practitioner during surgery times do you?

|  | All the time | Some of the time | Occas-ionally | Never |
| --- | --- | --- | --- | --- |
| Triage of GP attenders? |  |  |  |  |
|  |  |  |  |  |
| Phlebotomy? |  |  |  |  |
|  |  |  |  |  |
|  | All the time | Some of the time | Occasionally | Never |
| Carry out smear tests where applicable? |  |  |  |  |
|  |  |  |  |  |
| Carry out STD screening? |  |  |  |  |
|  |  |  |  |  |
| Assist in minor operations? |  |  |  |  |
|  |  |  |  |  |
| Advise on contraception? |  |  |  |  |
|  |  |  |  |  |
| Advise on asthma therapy? |  |  |  |  |
|  |  |  |  |  |
| Give vaccinations? |  |  |  |  |
|  |  |  |  |  |
| Carry out ECGs? |  |  |  |  |
|  |  |  |  |  |
| Remove sutures? |  |  |  |  |
|  |  |  |  |  |
| Carry out ear syringing? |  |  |  |  |
|  |  |  |  |  |

Have you any comments on the development of multi­disciplinary teams with the prison primary care system?

………………………………………………………………………………..

………………………………………………………………………………..

………………………………………………………………………………..

………………………………………………………………………………
